# Sensations of Cold

**Published:** 1892-04

**Authors:** 


SENSATIONS OF COLD in some parts of the body are sometimes produced by Rhus-tox., in some cases by Ammon-carb., and others by Ars. For a good selection it is necessary to observe the slightest moral or physical symptom.—*Revista Homœopatica,* April, 1891.

